# The Epidemiology and Trend of Cancer in Jordan, 2000–2013

**DOI:** 10.1155/2018/2937067

**Published:** 2018-10-17

**Authors:** Yousef S. Khader, Ghazi F. Sharkas, Kamal H. Arkoub, Mahmoud A. Alfaqih, Omar F. Nimri, Albaraa M. Khader

**Affiliations:** ^1^Faculty of Medicine, Jordan University of Science and Technology, Irbid 22110, Jordan; ^2^Ministry of Health, Amman, Jordan

## Abstract

**Objectives:**

This study aimed to determine the overall incidence, trend, and epidemiology of cancer among Jordanians from 2000 to 2013 using data extracted from Jordan's Cancer Registry (JCR).

**Methods:**

All cancer cases among Jordanians registered between 2000 and 2013 were analyzed using CanReg software and SPSS. The overall crude incidence rates (CIRs) and the age standardized rates (ASRs) of cancer per 100,000 were calculated.

**Results:**

A total of 58788 cancer cases were registered during the period 2000-2013. Of those, 28545 (48.6%) were males and 30243 (51.4%) were females. About three-quarters (77.3%) of the registered patients were ≥ 40 years in age. Overall, the average crude cancer incidence rate was 82.8/100,000 population during the 14-year study period. On the other hand, the ASR was 126/100,000 during the same period (124.2 /100,000 for males and 128.4 /100,000 for females). The cumulative top cancers among males were colorectal, lung, lymphoma, urinary bladder, and prostate, respectively, while those among females were breast, colorectal, lymphoma, thyroid, and uterine. The number of cancer cases has increased from 3370 in 2000 to 5409 in 2013 (60.5% increase over the 14 years). The percentage of increase was 68.4% in females and 52.5% in males. The ASR has also increased from 113.6 per 100,000 in 2000 to 142.1 per 100.000 in 2013 with a 25.1% of increase during the 14 years.

**Conclusion:**

Over the 14-year study period, incidence of cancer in Jordan has increased. However, it remains lower than that in other Eastern Mediterranean and Western countries. We recommend initiating screening programs for the most common types of cancer in Jordan that have valid screening tests to detect cancer during its early stages and reduce overall morbidity and mortality.

## 1. Introduction

Cancer is currently a global health problem and one of the leading causes of morbidity and mortality. Despite the ongoing global effort to prevent cancer, a 70% rise in cancer incidence is projected in the following two decades [1]. Globally in 2013, there were 14.9 million incident cancer cases and 8.2 million cancer related deaths [2]. Prostate cancer was the most common cancer among men, whereas breast cancer was the most common cancer among women [2]. While the incidence and mortality rates for most cancers are decreasing in the United States and many other Western countries, the incidence and mortality rates are both rising in developing countries [3]. Indeed, the diagnosis of new cancer cases in less developed countries is projected to increase from 56% of the world total cases in 2008 to more than 60% in 2030. This might be partially explained by the expected increase in life expectancy and population growth [3].

According to GLOBOCAN cancer estimation in 2012, about 57% of new cancer cases and 65% of the cancer deaths occurred in the less developed regions [4]. The overall age standardized cancer rate (ASR) in 2012 was almost 25% higher in men than in women, with rates of 205 and 165 per 100,000, respectively [4]. There was almost fivefold variation in male incidence rates across the different regions of the world on global level. The rates were ranging from 79 per 100,000 in Western Africa to 365 per 100,000 in Australia/New Zealand. In females, there was less variation in the incidence rates (almost threefold) with rates ranging from 103 per 100,000 in South-Central Asia to 295 per 100,000 in Northern America [4].

The top five cancers diagnosed worldwide among men in 2012 were lung cancer (16.7%), prostate cancer (15%), colorectal cancer (10%), stomach cancer (8.5%), and liver cancer (7.5%), whereas the top five cancers diagnosed in females were breast cancer (25.2%), colorectal cancer (9.2%), lung cancer (8.7%), cervix cancer (7.9%), and stomach cancer (4.8%) [5]. The most common incident cancers among females in 2015 were breast cancer, leukemia, and cervical cancer. The most common incident cancers in males in 2015 were tracheal bronchus and lung cancer, followed by prostate cancer and stomach cancer [6].

The Eastern Mediterranean Region (EMR) countries are experiencing marked variation in cancer incidence [6]. The incident cancer cases have increased by 46.1% between 2005 and 2015 [7]. The overall percentage change in the number of incident cancer cases ranged from 36.8% in Somalia to 182.9% in the United Arab Emirates [7]. The cancer incidence rates in the EMR were highest in Lebanon (204 and 193 per 100,000 in males and females, respectively) [8]. Six National Cancer Registries in the EMR (United Arab Emirates, Bahrain, Saudi Arabia, Oman, Qatar, and Kuwait) had reported 95,183 newly diagnosed cancer cases from January 1998 to December 2007. The average annual cancer incidences for the 10-year period from 1998 to 2007 were similar for GCC males and females (79.3 per 100,000). [9].

A study reporting cancer incidence rates from 2005 to 2010 in four countries of the Middle East Cancer Consortium (MECC) registries (Cyprus, Jordan, Israel, and Izmir (Turkey)) showed that Jordan has the lowest rates among both genders [10]. This study aimed to determine the overall incidence, trend, and epidemiology of cancer among Jordanians from 2000 to 2013.

## 2. Methods

All cancer cases diagnosed and registered among Jordanians for the period 2000-2013 were included. The data were requested officially from Jordan Cancer Registry (JCR) and were obtained in CanReg software format. Relevant variables for the purpose of the study were obtained including demographic data (gender, age) and tumor details (date of diagnosis, primary site, histology, behavior, grade, stage, and basis of diagnosis). The abstracted data were coded according to the international classification of disease ICD-9 or -10 morbidity and mortality coding system. The primary site (topography) and histology (morphology) of the malignancies are identified and coded according to the International Classification of Diseases for Oncology 3rd Edition (ICDO- 3), published by the World Health Organization (WHO) in 2000. Cases with a behavior code of 2 or 3 in the ICD-O-3 are included in the registry.

CanReg 4.31 software of the International Agency for Research on Cancer (IARC), Excel sheet, and SPSS version 20 were used to analyze the data. The crude incidence rates (CIRs), the age specific incidence rates (ASIRs), ASRs for each year, and the average ASR (calculated using the direct method of standardization and the world standard population to facilitate national and international comparisons) were all calculated.

## 3. Results

### 3.1. Distribution of Registered Cancer Cases

A total of 58788 cancer cases were registered during the period 2000-2013. Of those, 28545 (48.6%) were males and 30243 (51.4%) were females (male to female ratio = 0.94: 1).  Table 1 demonstrates the frequency distribution of these cases according to gender and age. About three-quarters (77.3%) of registered patients aged ≥ 40 years. Almost 1.4% of patients aged 10-14 years.

### 3.2. Cancer Incidence Rate

Overall, the average crude cancer incidence rate was 82.8/100,000 population during the 14-year period (76.1/100,000 for males and 89.9/100,000 for females). The ASR was 142.1/100,000 during the study period (137.5/100,000 for males and 147.5/100,000 for females).  Table 2 shows the average ASIRs during the 14-year period. Among males, the lowest average ASIR was in the age group 10-14 years (9.4/100,000) and the highest was in the age group 80-84 years (1029.8/100,000). Among females, the lowest average ASIR was in the age group 5-9 years (7.4/100,000) and the highest average ASIR was in the age group 75-79 years (664.5/100,000).

### 3.3. Top Cancers and Their Incidence Rates

The cumulative top ten cancers in males during the 14-year period were colorectal (12.3%), lung (11.6%), lymphoma (8.9%), urinary bladder (8.3%), prostate (7.9%), leukemia (7.0%), brain (4.4%), stomach (3.9%), larynx (3.6%), and kidney (2.7%) (Table 3). The average ASRs for the leading cancers per 100,000 males were 16 for colorectal cancer, 15.6 for lung cancer, 8.6 for lymphoma, 11.1 for urinary bladder, and 11.3 for prostate cancer (Table 3).

The cumulative top ten cancers in females were breast (35.6%), colorectal (9.6%), lymphoma (6.9%), thyroid (5.4%), uterus (5.1%), leukemia (4.9%), ovary (3.3%), brain (2.7%), stomach (2.3%), and lung (2.3%). The average ASRs for the leading cancers per 100,000 females were 45.3 for breast cancer, 13.2 for colorectal cancer, 7.4 for lymphoma, 5.3 for thyroid cancer, and 7 for uterine cancer (Table 3)

### 3.4. Trend of Cancer

**Figure 1:**
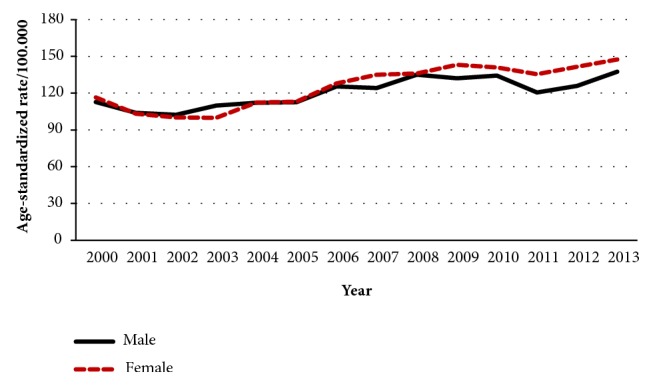
The trend in the age-standardized incidence rates of cancer for males and females in Jordan, 2000-2013.

**Figure 2:**
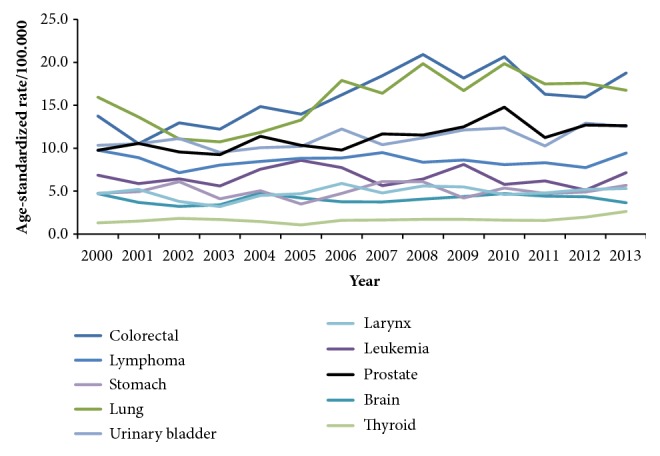
The trend in the age-standardized incidence rates of top 10 cancers for males in Jordan, 2000-2013.

**Figure 3:**
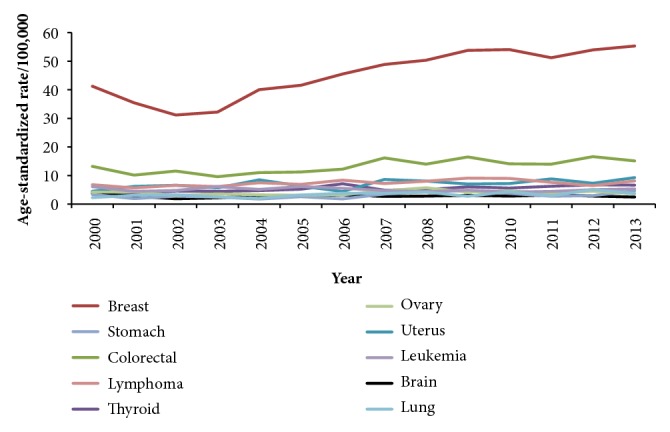
The trend in the age-standardized incidence rates of top 10 cancers for females in Jordan, 2000-2013.

The number of cancer cases has increased from 3370 in 2000 to 5409 in 2013 (60.5% increase over the 14 years). The percent of increase was 68.4% in females and 52.5% in males. The ASR has increased from 113.6 per 100,000 in 2000 to 142.1 per 100.000 in 2013 with a 25.1% of increase during the 14 years. Among males, the ASR has increased from 112.7 per 100,000 in 2000 to 137.5 per 100,000 in 2013 (Figure 1). Among females, the ASR has increased from 116.5 per 100,000 in 2000 to 147.5 per 100,000 in 2013 (Figure 1). The lowest ASR was in 2003 (99.5 per 100,000), and the highest was in 2013 (142.1 per 100,000). Figures 2 and 3 show the trend in the age-standardized incidence rates of top 10 cancers for males and females, respectively.

## 4. Discussion

This descriptive epidemiological study included all cancer cases registered in JCR in Jordan from 2000 to 2013. The start year was set to be 2000 because the data in JCR became more comprehensive and complete. According to the last published annual report of JCR in 2013, a total of 5416 new cancer cases were registered among Jordanians. The CIR of all cancers among Jordanians was 82.9 per 100,000 population (76.2 for males and 90.1 for females). The ASR was 142.1 per 100,000 population (137.4 for males and 147.5 for females).

There was a consistent debate that cancer in Jordan represents a major epidemic and that the incidence is unusually getting higher. In this study, the number of incident cancer cases over the 14-year period showed a steady increase (an overall 60.5% increase), but with no sharp increases. The rise was proportional to the natural population growth, with no evidence of epidemics all over the study period. According to the Global Burden of Disease 2015 Study, the incident cancer cases have increased by 46.1% between 2005 and 2015 in the EMR region. The overall percentage change in the number of incident cancer cases ranged between 36.8% in Somalia and 182.9% in the UAE. EMR countries with high income as well as Lebanon experienced the largest increase in cancer incidence [7].

On cumulative basis, cancer was slightly higher in females. This finding is consistent with the findings of some studies [11–14], while it is not consistent with findings of other studies [15–18]. This is most probably related to the high and increasing incidence of breast cancer in females. The female cancers predominance from 2006 onwards may be attributed to some extent to launching successful national campaigns for cancer awareness and early detection on female breast cancer. The Global Burden of Disease Study 2015 findings revealed that females had higher cancer incidence in 2015 than males, with CIR of 199.6/100,000 in females and 163.3/100,000 in males in the EMR countries including Jordan [7].

The number of cancer cases has increased with age, apparently at the age of 40 years and higher. More than two-thirds of the cases occurred between the 4th and the 7th decade. Thereafter, cases started to decrease and obviously declined at age of 80 years. This is mostly related to the survival bias and lower number of population after the age of 80 years as the life expectancy in Jordan is 72 years (73 years for males and 74 years for females).

In this study, the ASR of cancer in Jordan was 137.5 per 100,000 in 2013. In the global burden study [7], the ASR for cancer in Jordan was estimated to be 153/100,000 in 2015. The ASR was lower than that in the EMR (163/100,000) including countries such as Lebanon (284/100,000), Libya (226/100,000), Tunisia (224/100,000), UAE (213/100,000), and Iran (208/100,000). However, the rate in Jordan was higher than the rates in the gulf region including Qatar (144/100,000), Kuwait (139/100,000), Oman (121/100,000), Bahrain (134/100,000), and Saudi Arabia (104/100,000) [7]. The differences in cancer incidence between these countries might be related to the variable magnitude of risk factors of cancer including tobacco smoking, physical inactivity, unhealthy diet, and environmental pollution.

Globally, the highest ASRs of cancer are usually recorded in high income countries of North America, Western Europe, Japan, Korea, Australia, and New Zealand. Intermediate rates were recorded in Canada, South America, Eastern Europe, and many South Asian countries, and the lowest rates were recorded in many countries in Africa and South Asia [5]. High-income countries (HIC) are experiencing the highest incidence rates for all cancer sites, and especially for lung, colorectal, breast, and prostate cancer. Some low- and middle-income countries (LMIC) commenced to have higher rates as well. LMICs have the highest rates of stomach, liver, esophageal, and cervical cancer [19]. The relatively low cancer incidence in Jordan might be related to the well-established national programs for disease control, health promotion campaigns, and health education [11].

The differences in cancer rates between Jordan and developed or industrialized countries are usually attributed to the degree of modernization, and lifestyle changes including smoking, dietary habits, and physical activities. Fat consumption in Jordan does not reach the levels of industrialized societies and the traditional diet of Jordanians consists of a moderate intake of fruits and vegetables rather than meat [12].

Consistent with other studies [13–15], the overall trend of CIR and ASR of cancer showed a steady increase between 2000 and 2013. This was also consistent with a population based cancer registry study in Setif, Algeria, between 1996 and 2010, where the overall cancer incidence increased significantly in both men and women [16].

In this study, slight differences between genders were detected in the percent of change in CIR and ASR from 2000 to 2013. It was higher in females (28% and 26.6%, respectively) than males (19.5% and 22%, respectively). Similar finding has been reported by another study [17]. There were no sharp increases in CIR and ASR rates either in males or in females over the studied period. The average ASR of cancer was 120.6/100,000 among males in this study which was lower than the rates reported in Egypt Garbieh Cancer Registry (137.1/100,000, 2003-2007), Bahrain Cancer Registry (142.1/100,000, 2003-2007), Qatar Cancer Registry (154.7/100,000, 2003-2007), Bengazi Cancer Registry (154.7/100,000, 2003-2007), and Tunisia Cancer Registry (129.9/100,000, 2003-2007), but was higher than the rate reported in Kuwait Cancer Registry (94.1/100,000, 2003-2007) and Saudi Cancer Registry (104.1/100,000, 2003-2007) [18].

The average ASR of cancer was 125.2/100,000 among females in this study which was lower than that reported in Egypt Garbieh Cancer Registry (125.1/100,000, 2003-2007), Bahrain Cancer Registry (150/100,000, 2003-2007), and Qatar Cancer Registry (151.1/100,000 2003-2007), but was higher than the rate reported in Benghazi Cancer Registry (110/100,000, 2003-2007), Tunisia Cancer Registry (99.7/100,000, 2003-2007), and Saudi Cancer Registry (103.9/100,000, 2003-2007) [18].

The ASRs per 100,000 in western countries were much higher than the values in Jordan and Arab countries; for example, the rates reached as high as 438.7 for males and 332.6 for females in Belgium (2004-2007), 447.4 for males and 350.2 for females in Ireland (2003-2007), 327.1 for males and 276.1 for females in the Netherlands (2003-2007), 358.4 for males and 301.5 for females in the UK (2003-2007), 335.0 for males and 273.6 for females in Canada (2003-2007), and 363.4 for males and 284.6 for females in USA, the National Program of Cancer Registries (NPCR) (42 states) (2003-2007) [18].

The most frequent cancers on cumulative basis in both genders were breast, colorectal, lymphoma, lung, and leukemia. This was consistent with the world top cancers as far as lung, breast, and colorectal cancer are concerned according to GLOBOCAN estimates 2012 [4] and also consistent with the EMR top cancers in the global cancer burden study 2015 as far as breast cancer, lung cancer, and colorectal cancer are concerned. Furthermore, colorectal cancer was the second most frequent incident cancer in 2015 in Jordan, Kuwait, Lebanon, Libya, Qatar, and Saudi Arabia [7]. In the Gulf Cooperation Council states (United Arab Emirates, Bahrain, Saudi Arabia, Oman, Qatar, and Kuwait), advanced breast cancer, colorectal cancer, leukaemia, thyroid cancer, and non-Hodgkin lymphomas were the most common cancers affecting younger populations compared with other countries. At least two of the top cancers in Jordan were in common with the most frequent cancers in GCC states, namely, breast cancer and colorectal cancer [20].

The cumulative leading cancers in males during the study period were colorectal, lung, prostate, urinary bladder, lymphoma, leukemia, stomach, larynx, brain, and thyroid cancers. GLOBOCAN estimates figured lung, prostate, colorectal, stomach, liver, bladder, esophagus, NHL, kidney, and leukemia as the top globally diagnosed cancers among men in 2012 [4] and the world cancer report 2014 stated that the five most common globally diagnosed male cancers were lung, prostate, colorectal, stomach, and liver cancers [5]. Upon comparison, there was coherence between several leading male cancers in Jordan compared with the global cancers except for larynx, brain, thyroid, liver, esophagus, and kidney cancers.

The aforementioned discussion stresses the burden of certain cancers particularly breast, colorectal, and lung on the global level and the regional level and in Jordan as well.

The cumulative top ten cancers in females during the study period were breast, colorectal, lymphoma, uterus, thyroid, leukemia, ovary, lung, stomach, and brain. GLOBOCAN estimates showed that the top globally diagnosed cancers among women in 2012 were breast, colorectal, lung, cervix, stomach, corpus uteri, ovary, thyroid, liver, and NHL [4]. According to the world cancer report 2014, the five most frequent cancers among women were breast, colorectal, lung, cervix, and stomach [5]. Upon comparison, there was coherence between several leading female cancers in Jordan compared with the global cancers except for leukemia, ovary, cervix, liver, and brain cancers.

Cervical cancer was not among the leading cancers in Jordanian females. A cancer trend study was performed about cervical cancer in Jordan from 2000 to 2013. The study revealed that the average age standardized rate (ASR) was 2.0/100,000 women. Over the 14-year period, the ASR for cervical cancer has decreased by 28.6% from 2.1 per 100,000 women in 2000 to 1.5 per 100,000 women in 2013 [21]. The reason for that may be related to the low prevalence of risk factors of cervical cancer in Jordan including multiple sex partners.

This study explored the longest time trend for cancer incidence in Jordan, which enables the best comparison of more representative period of time, and utilized the best of JCR data, where the quality of registered data became more accurate and detailed starting from the year 2000. The detailed and comprehensive analyses for the incidence of cancer, cancer trends, and top ten cancers have resulted in various and informative results.

In conclusion, cancer in Jordan was increasing steadily all over the period 2000-2013. The incidence of cancer was lower than that in the EMR and western countries. Thorough investigations are recommended to identify the reasons behind the persistent occurrence of certain cancers among Jordanian males and females. It is of value to conduct cost-effectiveness studies and subsequent implementation of national cancer screening programs for the commonest cancers in Jordan for early detection to reduce morbidity and mortality. It is of benefit to work on already known risk factors of cancer currently prevailing among Jordanians including tobacco smoking, obesity, physical inactivity, pollution, and unhealthy diet.

## Figures and Tables

**Table 1:** The distribution of cumulative cancer cases registered in Jordan in the period 2000-2013 according to gender and age.

Age group	Gender				Total (N = 58788)	
	Male (n = 28545)	%	Female (n = 30243)	%	N	%
0-4	719	2.5	546	1.8	1265	2.2
5-9	511	1.8	360	1.2	871	1.5
10-14	459	1.6	357	1.2	816	1.4
15-19	604	2.1	560	1.9	1164	2.0
20-24	664	2.3	709	2.3	1373	2.3
25-29	760	2.7	977	3.2	1737	3.0
30-34	999	3.5	1644	5.4	2643	4.5
35-39	1183	4.1	2226	7.4	3409	5.8
40-44	1585	5.6	2969	9.8	4554	7.7
45-49	1881	6.6	3190	10.5	5071	8.6
50-54	2334	8.2	3158	10.4	5492	9.3
55-59	2799	9.8	3112	10.3	5911	10.1
60-64	3650	12.8	3327	11	6977	11.9
65-69	3752	13.1	2758	9.1	6510	11.1
70-74	3122	11.0	2123	7.0	5245	8.9
75-79	1997	7.0	1203	4.0	3200	5.4
80-84	976	3.4	687	2.3	1663	2.8
85-120	550	2.0	337	1.1	887	1.5

**Table 2:** Age specific incidencee rates of cancer among males and females in Jordan, 2000-2013.

Age group	Male				Female				Total			
	N^*∗*^	Av /N^*∗∗*^	%	Av/ASIR^+^	^*∗*^N	*◆*Av/N	%	°Av/ASIR	^*∗*^N	*◆*Av/N	%	°Av/ASIR
0-4	719	51	2.5	13.9	546	39	1.8	11.1	1265	90	2.2	12.5
5-9	511	37	1.8	10.0	360	26	1.2	7.4	871	62	1.5	8.7
10-14	459	33	1.6	9.4	357	26	1.2	7.7	816	58	1.4	8.6
15-19	604	43	2.1	13.5	560	40	1.9	13.3	1164	83	2.0	13.4
20-24	664	47	2.3	15.3	709	51	2.3	17.5	1373	98	2.3	16.4
25-29	760	54	2.7	20.4	977	70	3.2	29.1	1737	124	3.0	24.5
30-34	999	71	3.5	30.9	1644	117	5.4	55.2	2643	189	4.5	42.6
35-39	1183	85	4.1	45.2	2226	159	7.4	92.1	3409	244	5.8	67.8
40-44	1585	113	5.6	82.1	2969	212	9.8	162.9	4554	325	7.7	121.3
45-49	1881	134	6.6	138.6	3190	228	10.5	246.5	5071	362	8.6	191.3
50-54	2334	167	8.2	231.9	3158	226	10.4	319.7	5492	392	9.3	275.4
55-59	2799	200	9.8	322.3	3112	222	10.3	345.9	5911	422	10.1	334.3
60-64	3650	261	12.8	450.3	3327	238	11.0	458.5	6977	498	11.9	454.2
65-69	3752	268	13.1	650.4	2758	197	9.1	513.7	6510	465	11.1	584.5
70-74	3122	223	10.9	851.9	2123	152	7.0	590.6	5245	375	8.9	722.5
75-79	1997	143	7.0	1016.2	1203	86	4.0	664.5	3200	229	5.4	847.6
80-84	976	70	3.4	1029.8	687	49	2.3	587.3	1663	119	2.8	785.3
85-120	550	39	1.9	893.1	337	24	1.1	474.6	887	63	1.5	669.0

^*∗*^N = total number of cases in the age group during the 14-year period.

^*∗∗*^ **Av/N:** the average number of cases in the age group during the 14-year period.

+**Av/ASIR**= the average ASIR in the age group during the 14-year period.

**Table 3:** The cumulative top ten cancers in males and females, Jordan, 2000-2013.

Male			Female			Total		
Primary Site	N	%	Primary Site	N	%	Primary Site	N	%
Colorectal	3532	12.3	Breast	10780	35.6	Breast	10957	18.6
Lung	3332	11.6	Colorectal	2912	9.6	Colorectal	6444	10.9
Lymphoma	2546	8.9	Lymphoma	2083	6.9	Lymphoma	4629	7.9
U.bladder	2384	8.3	Thyroid	1623	5.4	Lung	4033	6.8
Prostate	2267	7.9	Uterus	1532	5.1	Leukemia	3484	5.9
Leukemia	2008	7.0	Leukemia	1476	4.9	U.bladder	2723	4.6
brain	1263	4.4	Ovary	986	3.3	Prostate	2267	3.8
Stomach	1129	3.9	Brain	833	2.7	Thyroid	2112	3.6
Larynx	1040	3.6	Stomach	712	2.3	Brain	2096	3.6
Kidney	772	2.7	Lung	701	2.3	Stomach	1841	3.1
Others	8331	29.1	Others	6668	22.0	Others	18324	31.1

Total	28604	100.0	Total	30306	100.0	Total	58910	100.0

## Data Availability

The data used to support the findings of this study are available from the corresponding author upon request.
